# Contextual Action Cues from Camera Sensor for Multi-Stream Action Recognition

**DOI:** 10.3390/s19061382

**Published:** 2019-03-20

**Authors:** Jongkwang Hong, Bora Cho, Yong Won Hong, Hyeran Byun

**Affiliations:** Department of Computer Science, Yonsei University, Seoul 03722, Korea; jkhong9@yonsei.ac.kr (J.H.); chobora@yonsei.ac.kr (B.C.); yhong@yonsei.ac.kr (Y.W.H.)

**Keywords:** action recognition, contextual information, multi-stream fusion

## Abstract

In action recognition research, two primary types of information are appearance and motion information that is learned from RGB images through visual sensors. However, depending on the action characteristics, contextual information, such as the existence of specific objects or globally-shared information in the image, becomes vital information to define the action. For example, the existence of the ball is vital information distinguishing “kicking” from “running”. Furthermore, some actions share typical global abstract poses, which can be used as a key to classify actions. Based on these observations, we propose the multi-stream network model, which incorporates spatial, temporal, and contextual cues in the image for action recognition. We experimented on the proposed method using C3D or inflated 3D ConvNet (I3D) as a backbone network, regarding two different action recognition datasets. As a result, we observed overall improvement in accuracy, demonstrating the effectiveness of our proposed method.

## 1. Introduction

**Figure 1:**
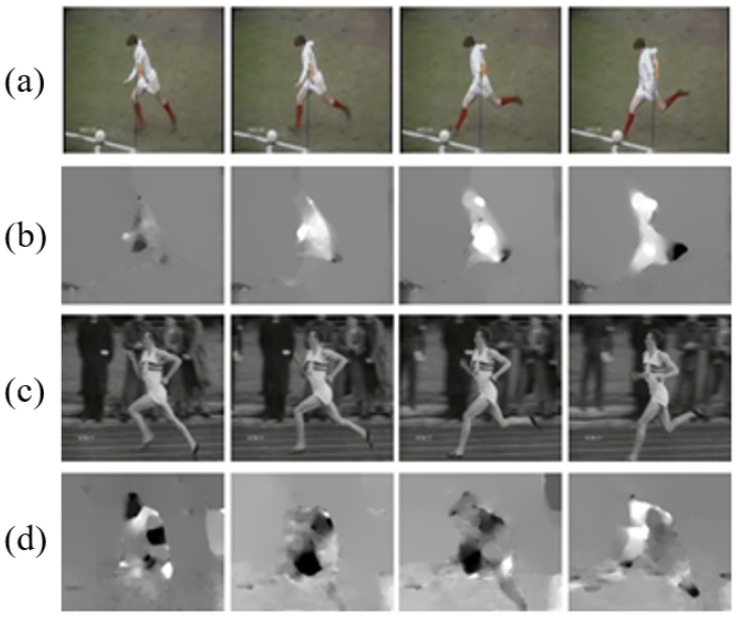
Examples from UCF sports [11] dataset, each row representing the image sequence of the video. (**a**) RGB images from the “Kicking” class. (**b**) Corresponding optical flow images to (**a**). (**c**) RGB images from the “Run” class. (**d**) Corresponding optical flow images to (**c**).

With the recent advent of deep learning, visual sensor-based action recognition technologies are being actively researched and used in a wide range of applications, e.g., person activity analysis [1], event detection [2], and video surveillance systems [3]. Most of the previous action recognition studies used video data as the source of temporal and spatial information, as opposed to static images containing only spatial information captured from camera sensors. Therefore, it is necessary to develop the way to incorporate and utilize both temporal and spatial information of video clips effectively. These two types of information, the appearance information and optical flows, are fully utilized by adopting the two-stream networks [4]. However, there is a limitation to using only two types of time and space information, as shown in Figure 1. To solve this problem, there were previous studies [5,6,7,8] that additionally used contextual information to improve the accuracy of action recognition. Contextual information refers to information that can be deduced in addition to high-level temporal and spatial information, and can be more semantic and abstract [9,10].

The two pictures of Figure 1 are RGB and optical flow images corresponding to the classes of “Running” and “Kicking”, respectively. Previous action recognition studies aimed to distinguish between these actions only with the appearance information and the optical flows. However, as shown in Figure 1, it is not easy to distinguish between the two actions by only the RGB and the optical flow information, due to their similarity of appearance and movement. Otherwise, if the action recognition model can capture additional object “ball” and utilize the contextual information in the frame, these actions can be distinguished more accurately. Thus, in addition to the temporal and spatial information, the proposed method utilizes contextual information such as correlation of multiple objects (e.g., singers and people around them) and action entities. In addition, we propose a method to use extracted pose information from images for action recognition. Pose information is important in action recognition, which is valuable in two aspects. First, unlike RGB information, which is high-dimensional information, it can convey clear and certain information. Second, actions share typical global poses, which can help to distinguish different actions. Unlike RGB or optical flow, which is sensitive to small changes or background clutters, pose information is robust because it is shared between action categories. In summary, in this paper, we propose to use specific, clear action information as an additional input, unlike high-dimensional, abstract visual information. Specifically, we trained two additional 3D convolutional neural networks using pose and pairwise information. Especially for pairwise information, we propose a method to construct the relationship between action entities and related objects, as well as the definition of interaction, because there are no annotations for action relationships in action recognition datasets.

The main contributions of this work are five-fold:

1. In addition to the two streams (RGB and optical flow), we propose to use additional pose and pairwise streams to improve the performance of action recognition.
2. Our proposed pairwise stream does not require related object and interaction annotations. To construct a pairwise stream, object detection and interaction recognition are required. Datasets for existing human-object interaction (HOI) studies should be trained using a fully-annotated dataset with the related object location and interaction label. Furthermore, the candidates of the interaction between object and human are all pre-defined. However, in our work, we modeled a pairwise stream in action recognition datasets, for which such annotations are not available.
3. Instead of using the bounding box-based approach, we suggest using a mask-based pairwise stream. We show that the mask-based pairwise stream further improves the performance.
4. We propose the pose stream, which can deliver explicit and robust information for classifying the action category.
5. The proposed method outperforms well-known C3D-[12] and state-of-the-art inflated 3D ConvNet (I3D)-based [13] results.

The composition of this paper introduces the relevant research in Section 2 and the proposed method in Section 3. Section 4 introduces the experimental settings, and Section 5 includes the results of the proposed method and the analysis of the results and finally concludes in Section 6.

## 2. Related Work

### 2.1. Action Recognition

Action recognition studies are divided into hand-crafted features and features based on deep learning. Hand-crafted features are typically extracted from bag of words (BoW) [14] and dense trajectory [15]. Information on the shape of the space area is extracted using the histogram of oriented gradients (HOG) [16]. Furthermore, the information in the time domain is extracted via the optical flow [17]. However, this approach is not only costly to compute, but also limited to extracting features that contain low levels of information. To overcome these limitations, convolutional neural network (CNN)-based research is introduced to action recognition after active research on image-based deep learning. Two-stream networks [4] are the typical structures that are directly influenced by image-based architecture. The two-stream network is divided into a spatial stream and a temporal stream. The spatial stream analyzes spatial information of an RGB image. The temporal stream analyzes the time axis characteristics by using the optimal flow with motion information as the input. TSN [18] takes the multiple input segments from single videos and is able to capture the different scope of action in the timeline to improve the performance of [4]. In order to learn the temporal relationship between these visual features, there are some studies that have used 2D-based CNN features such as long short-term memory network (LSTM) [19] inputs corresponding to the temporal order [20,21]. However, since the size and length of the video datasets become longer, their recognition performances become limited. This is because the LSTM is inefficient at dealing with the long input sequences. Accordingly, the 3D convolution neural network (3D-CNN) [12] is proposed, which adds a time axis to the 2D convolution that allows both spatial and temporal information to be learned at the same time and is very suitable for analyzing video image data. However, increasing the convolutional kernel dimension yields a considerable increase of the parameters, making it difficult to train the whole network with a limited dataset size. P3D [22], R(2+1)D [23] mitigate the parameter size issue with projection the 3D convolution with a combination of 2D convolution and 1D convolution, having less parameters compared to 3D convolution. Inflated 3D ConvNet (I3D) [13] approaches the parameter size issue differently. Instead of sacrificing the parameter size, it utilizes the image-based pre-trained weights in 3D convolution with even a deeper network of C3D [12] and is now the state-of-the-art in the field of action recognition, further enhancing the performance of action recognition.

### 2.2. Action Recognition Using Human Pose

A pattern of body movements shapes a person’s action, so their posture is one of the most important visual information in action perception. Thus, the number of studies that estimate information about a person’s posture and recognize their action through these pose characteristics has increased [5,6,7]. They used CNN to extract keypoints or body-part segmentation information that contains the location of an important human body part and used it as an input to recognize the action. Multi-region two-stream RCNN [5] is a typical study that characterizes a person’s body posture. Multi-region two-stream RCNN improves the performance of action recognition by analyzing a person’s body parts as four parts, each in its entirety, “upper half”, “bottom half”, and the entire wide area. Subdividing the body parts helped to distinguish ambiguous actions by analyzing the body’s vertical structure when recognizing them. In addition, the work in [6,7] proposed that posture estimation and action recognition should be conducted simultaneously, and the work in [7] consisted of an end-to-end structure that uses the time-to-space information of a person’s posture to help with action recognition. This end-to-end structure improved the performance of action recognition by using posture information of the person who is the subject of the action as vital information to recognize the person’s posture and the temporal flow of action through the 3D network.

### 2.3. Human-Object-Interaction

The human-object interaction (HOI) study has been progressing for decades. The primary HOI dataset is HICO [24] (Human Interaction with Common Objects), a set of large image data that includes subjects, objects, and relationships. In addition, the HICO-DET [8] dataset reinforced the HICO dataset by additionally annotating the detection information. Both datasets are used in the HOI [8] detection to detect the positions of subjects and objects. The other HOI-related dataset is the Verb-in COCO dataset [25], which is based on the MS-COCO dataset [26]. In the human-object interaction, a person’s action class is defined as more granular than the general action recognition dataset, due to various relationships between the action and different objects in images. This calls for a deeper understanding of a person’s action and the objects existing around them. In the field of HOI, principally, the subject of the action and the relationship with the objects associated with it are obtained through the location relationship between the two. HO-RCN [8] consists of three streams that recognize the relationship between a person and an object and also analyze the location relationship in the last stream. Furthermore, the work in [27] proposed a network architecture that pre-learns the distribution of location relationships between the subject and the object to infer the extent to which the object might exist. Research in these areas demonstrates the impact and importance of the relationship between a subject and object that affects the action.

## 3. Proposed Method

**Figure 2:**
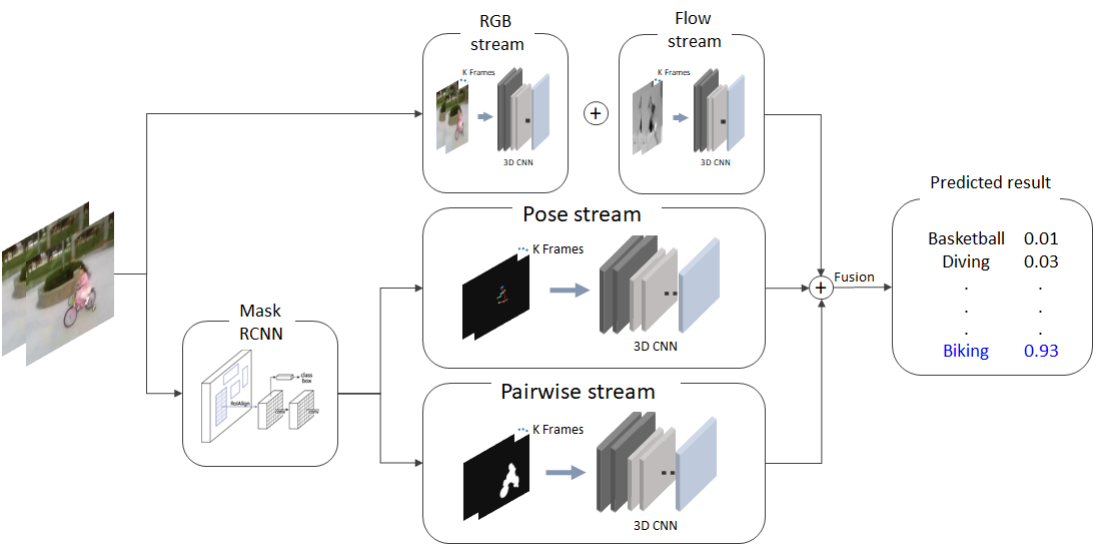
Overall architecture of the proposed method.

The networks proposed in this paper are as follows. Figure 2 shows the multi-stream network that uses all the given information. The overall network is divided into two parts: (1) the RGB/flow stream to process appearance and motion information; (2) the pose stream and pairwise stream to utilize contextual cues from features. Each stream uses the same structure of the backbone network (e.g., C3D [12] or I3D [13]). RGB: image, flow: optical flow, pose: keypoints image, and finally, pairwise: mask image, including the location relationship between subject and objects, are used as input. The keypoints image and pairwise mask image are obtained from the pre-trained Mask-RCNN [28] model. The key assumption of our proposed method is that information about an object (e.g., a person’s body posture, the object’s local contexts) has important cues for defining the subjects’ action.

### 3.1. RGB/Flow Stream

The action recognition network consists of convolutional neural networks to process each input stream, from image-based two-stream networks to 3D convolutional networks. In this paper, we adopted the widely-used 3D convolutional networks as our backbone network, to understand the meaning of spatial-temporal construction for each stream. Among 3D convolutional networks, C3D [12] showed the first practical performance of a 3D convolutional network in action recognition, and I3D [13] achieved the state-of-the-art performance at the current UCF-101. In the RGB stream, an RGB image is used as an input to recognize the action. The RGB information contains spatial information of background and objects that are all regarded as appearance information. In the flow stream, an optical flow [17] image is used as an input, which is extracted from a sequence of RGB images. In the flow stream, since optical flow depicts the frame difference between RGB images, so an optical flow image is regarded as motion information. We used the following cross-entropy loss for training both streams individually, as in Equation (1).

(1) $$loss=-\sum_{c=1}^{M}y_{i}\log\left(y_{i}^{\prime }\right)$$

where *M* stands for the number of the classes, $y_{i}$ is correctness of the $i^{\mathrm{th}}$ observation, and $y_{i}^{\prime }$ is the predicted probability of the $i^{\mathrm{th}}$ observation.

### 3.2. Pairwise Stream

This section proposes a pairwise stream that characterizes the relative position of a person and an object to understand the relationship between them. With mask-segmentation information obtained from Mask-RCNN [28], the pairwise stream extracts the characteristics of the spatial relationship between the subject of the action and the surrounding objects. Since the inputs of the pairwise stream focus only on the spatial relationship between people and the surrounding objects, the single mask image representing the position of the person and the object is used as an input to the pairwise stream instead of the pixel values of the image.

**Figure 3:**
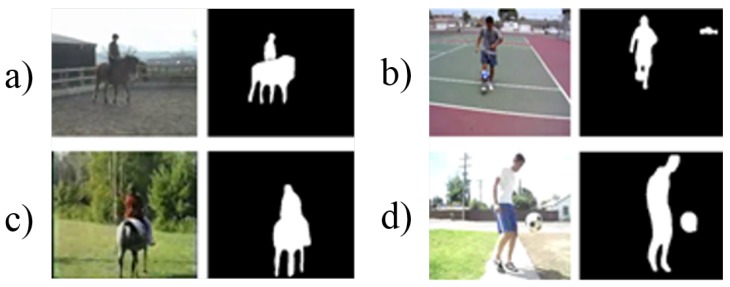
Example of an RGB image with corresponding pairwise inputs. (**a**) “HorseRiding”. (**b**) “SoccerJugglings”. (**c**) “HorseRiding”. (**d**) “SoccerJugglings”.

**Figure 4:**
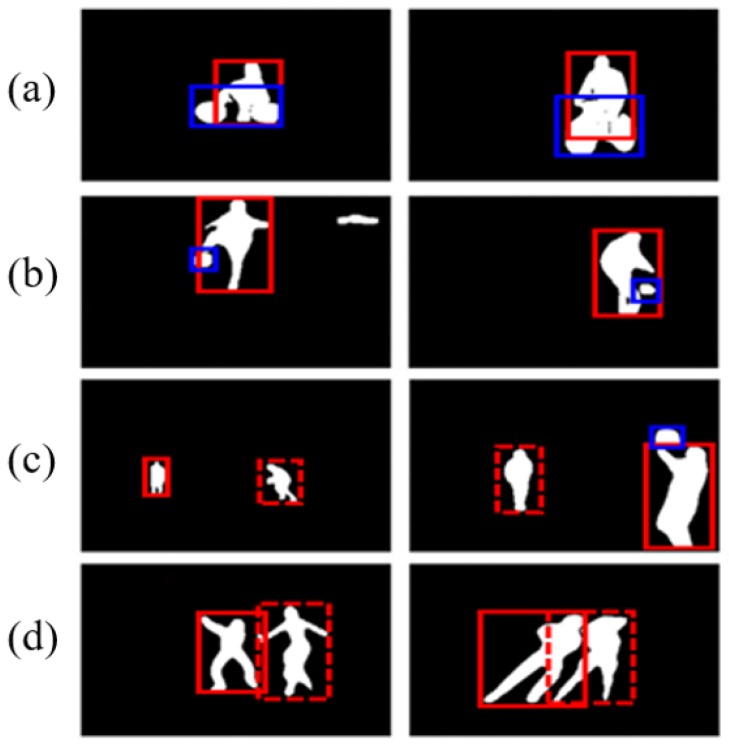
Details about pairwise stream inputs; images of each row are from the same video in different timelines. Solid red line boxes represent the “actors” in the action. Dotted red line boxes represent the “person” who is regarded as an “object”. Solid blue line boxes represent the “object” of “actors”. (**a**) “Biking”. (**b**) “SoccerJugglings”. (**c**) “Basketball”. (**d**) “IceDancing”.

The majority of HOI [8] studies use the dataset annotated with the relationship between action entities, objects, and the relationship, but action recognition datasets do not have such pre-defined ground truth annotation. Therefore, we propose the following protocol to construct the relationship between action entities and related objects, as well as the definition of the interaction for a pairwise stream. The first issue of constructing the relationship pair is that there is no pre-defined set of objects in the video dataset. Therefore, the “object” list required for the pairwise stream uses the class of the MSCOCO dataset [26] in the same way as the list of objects used in HOI [8]. The MS COCO dataset, a total of 80 classes, contains most of the objects associated with the UCF-101 dataset, such as “person”, “cycles”, “boat”, “dog”, “skis”, and “sports ball”. Meanwhile, the work in [8,25] used bounding boxes, but the proposed method used a mask as the input to provide further specific and rich relationship information. The differences between the mask and bounding boxes are further addressed in Section 4 with the comparison result. The second issue is the way of filtering the pair to reduce the noise to the stream. It is a strong assumption that every detected relationship is dedicated to defining the action. First, masks classified as “people” are regarded as action subjects, and other masks are classified as “objects”. Relationships tend to exist between entities and surrounding objects, so we only consider the context information of entities and objects within a predefined distance (150 pixels), ignoring distant objects that may be noise in defining the class. This distance is obtained through some preliminary experiments. The distance is calculated by the pixelwise distance between the mask centroid of the “person” and the “object”. The third issue is a frame with irrelevant objects. In this case, the other “person” that exists is considered as an object since this implies the group interaction to define actions, such as “diving” and “ice dancing”. Only the mask images corresponding to the previously-described conditions are used as input. The combined masks of the “person” and “object” pair are used as interaction information. Although this is not as explicit an interaction as HOI [8], it defines the implicit interaction of the dataset by the distance and location between the subject of the action and the object to use. Figure 3 shows examples of inputs to the pairwise stream. All the appearance information is excluded as the background, and only location and shape information of pair, people, and objects is used. Finally, there is a difference between the image-based and video-based methods in the definition of interaction. In the image-based methods, interactions in each image are various, but in a video-based scenario, it requires the video-level definition for global interaction, which differs from the frame-level definition. To define the video-level interaction pair, the action subject and related objects in each frame are the candidates of a video-level subject-objects pair. There are a couple of different cases to define the video-level pair interaction. Case 1: When the objects associated with the action are clear. For instance, most objects called “bicycle” and “ball” are detected in “Biking” and “SoccerJugglings”, respectively. Therefore, defining the video-level interaction of “Biking” for the (“bicycle”, “person”) pair and “SoccerJugglings” for the (“ball”, “person”) pair are clear. The first and second rows are classes of “Biking” and “SoccerJugglings” in Figure 4. Case 2: When the objects related to the action are not clear. Examples of these cases are the “Basketball” and “IceDancing” classes in the third and fourth rows of Figure 4. Most of the frames in those classes are without related significant objects. In this case, we first consider the actors–person interaction where the people around actors are the input objects related to considering the social interaction. If none of the major interactions exist, existing human–object and human–human interactions will be considered as an action. In Figure 4, the subject and object are marked with red and blue boxes. Furthermore, “actors” and “person” used as related objects are shown in red solid lines and dotted boxes, respectively.

### 3.3. Pose Stream

**Figure 5:**
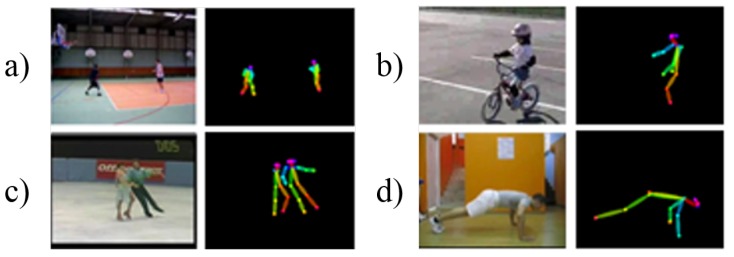
Example of an RGB image with corresponding keypoints inputs. (**a**) “Basketball”. (**b**) “Biking”. (**c**) “IceDancing”. (**d**) “HandbandPushups”.

In the pose stream, keypoints images are used as inputs. Keypoints images contain information about posture which is the connected key body parts of a person. These images are extracted via Mask-RCNN [28]. Figure 5 shows sample pairs of the RGB image and corresponding keypoints image obtained from the UCF-101 dataset [11]. The estimated keypoints images contain information about each person’s body part by highlighting joints and also depict one’s posture by connecting the pre-defined common connection, which infers one’s action. The pose stream shares the same manner of input settings as the RGB stream. The only difference between them is that clips of keypoints frames are used as input to the 3D network and optimize the same loss function, as described in Equation (1), for action recognition, since the goal of the stream is the same as RGB.

### 3.4. Multi-Stream Fusion

In this paper, each stream is trained individually, and for the final result, the final score *Y* is obtained from a weighted sum of scores as follows. Based on the late fusion method used by most two-stream action recognition algorithms, this paper obtained the final results through the following equation,

(2) $$Y_{score}=\alpha y_{RGB}^{\prime }+\beta y_{Flow}^{\prime }+\gamma y_{Pose}^{\prime }+\delta y_{Pairwise}^{\prime }$$

where $y^{\prime }$s are raw scores from corresponding stream outputs. Furthermore, α,β,γ,δ are numeric parameters. We determined the optimal parameter values, which showed the best performance, through a grid searching method.

## 4. Experiments’ Settings

### 4.1. Datasets

We evaluated our model on the UCF-101 dataset [11] and HMDB-51 dataset [29] in this paper. The UCF-101 consists of 13,320 action videos with 101 action classes and recorded under various human posture, camera movement, and backgrounds. The action classes are grouped into 25 groups, and each group contains up to seven videos. Each video has a resolution of 320 × 240. The HMDB-51 dataset [29] consists of 51 action classes of real-life video footage collected primarily from movies. Each class consists of at least 101 videos. Each video has a resolution of 320 × 240 and 30 fps. Both datasets have the number of different dataset split with the shared list of videos. In this paper, the experiments used “split 1”, and other results of compared methods were also the results of using “split 1”, for a fair comparison.

### 4.2. Networks

We use C3D [12] or I3D [13] as the backbone network architecture in our experiments. The input image size of each stream is 224 × 224 for I3D [13] and 112 × 112 for C3D [12]. All streams except flow stream use a three-channel input, while flow stream uses two channels. For I3D [13], the batch size is six input clips where each clip consists of 64 frames. We trained the network for 40 epochs. In addition, both the baseline experiment and proposed methods were trained from Kinetics [30] pre-trained model for a fair comparison between the additional contextual cues and external data. In the case of C3D [12], the batch size is 20, and the input clip size is 16 frames, while there are 30,000 steps for training the network. Both the baseline experiment and proposed methods in the network were trained from Sports-1M [31] for a fair comparison. All other hyperparameters for the training were identical with [12,13]. For multi-stream, I3D [13] backbone stream weights were α:0.5,β:0.7,γ:0.2, and δ:0.5. For C3D, since [12] uses only RGB steams, we regard RGB as backbone stream unlike I3D and add pose and pairwise streams to explore the impact of contextual information. The number of epochs was selected to satisfy enough epochs for the learning (the minimum epoch that converged with accuracy) through several preliminary experiments. The frame rate and batch size were set considering our GPU memory. Other parameters required for training were the as same as the parameters in the reproduction source code and the original papers of C3D and I3D.

The experiments were conducted on a server with Xeon Processor E5-2600 CPU and two NVidia GeForce GTX TITAN X GPUs. Training C3D and I3D required three days and five days, respectively, and the test time (response time) was less than 1000 milliseconds for both.

## 5. Results and Analysis

### 5.1. Mask vs. Bounding Box

Existing contextual cue-based recognition studies [25] used the bounding box as the contextual cues and evaluated the overall accuracy. However, the dataset used in this paper did not include annotated bounding boxes. Taking advantage of using Mask-RCNN, we compare the overall accuracy between using the bounding box and mask as the input of the pairwise stream in Table 1 with the I3D backbone network. When using masks as the input, it was 26.22% higher than bounding box based, and the combined result of all streams was 0.19% higher. We believe the performance difference was caused by the amount of information difference between the bounding box and the mask. The bounding boxes only contain location information, while masks contain shape information of the object and implicit posture information of the actor’s body. Furthermore, actor’s shape information is close to the pose stream’s keypoints image, but the experiment, Table 2, showed that keypoints information was more explicit than just shape information.

### 5.2. Results

Table 2 is the result of using C3D [12] and I3D [13] as backbone networks for the UCF-101 and HMDB-51 datasets. The results of the C3D [12] baseline (RGB only) were 84.26% and 80.35% for the pose stream and 79.97% for the pairwise stream. In the case of the C3D-based final multi-stream fusion, the result was 91.04% with a 6.84% performance improvement over the baseline. The baseline results for I3D in UCF-101 were 94.69% for RGB stream, 94.14% for flow stream, 69.15% for pose stream, and 76.02% for the pairwise stream. In multi-stream fusion, the two-stream RGB and flow baseline was 97.33%; while the addition of pose stream increased the accuracy by 0.56% and the addition of pair stream increased it by 0.13%, respectively. The HMDB-51 result also featured 0.78% and 0.26% improvement on the pose stream and pairwise stream. The final four stream results in both datasets were the following: 98.02% for UCF-101 and 80.92% for HMDB-51. The proposed method yielded improvement on both datasets from each baseline: 0.69% for UCF-101 and 0.85% for HMDB-51, which implies that the contextual information was not bounded by specific datasets. Please note that even though the absolute value of the improvement was slight, the baseline performances were already high (97.33% and 80.07%), so improving beyond them was challenging and worthwhile. Adding the different types of contextual streams (e.g., pose, pairwise) unanimously improved the overall results, which also implies that none of the contextual streams shared the same contextual information.

Table 3 is the result of the UCF-101 dataset class accuracy of the baseline and proposed method. From the top, the most improvement of accuracy in classes is listed in order. The baseline is a combination of the RGB and flow of I3D networks (97.33% in Table 2), and the proposed method is the performance of adding both pose and pairwise streams to the baseline (98.02% in Table 2). We included whether the contextual information improved or measured equally the accuracy in a total of 100 classes over the baseline. The improvements of each class were varied from 14.29%–2.27% accuracy. In particular, the “HandbandPushups” and “HandStandWalking” classes showed significant improvement of 14.29% and 8.82%, respectively, compared to other classes. Most of the classes with improved accuracy contained objects that were related to an action or had a clear posture in actions where RGB and flow images alone were hard to capture.

### 5.3. Comparison with Existing Methods

Table 4 is a comparison of the proposed method with other existing methods on the UCF-101 dataset and the HMDB-51 dataset. Since conventional action recognition studies were based on the two-stream method, the following scores are two-stream based, except our proposed multi-stream contextual models, which include contextual streams (e.g., pose, pairwise) alongside the spatial-temporal stream. In Table 4, the top half portions were based on the Kinetics [30] pre-trained I3D-based model, and the bottom half portions were the Kintecits and ImageNet [32] pre-trained model, which held the state-of-the-art performance in UCF-101. Even though, our baseline model was based on [13], due to the hardware limitation and hyperparameter tuning, there was a little performance gap with [13] in both datasets. With the weights of the same network in both dataset, we believe the improvements of the proposed method were higher than what we posted in here. As Table 4 shows, the proposed method not only suppressed the counterpart of [13] by 0.42%, but also was on par with current state-of-the-art UCF-101 dataset performance, which is 98.0% with the additional ImageNet dataset for pre-training. Our model held a clear advantage over the addition of the ImageNet dataset. As we described before, our models’ inputs were all extracted from the same dataset images, while the ImageNet dataset was the clear external dataset, and also, the dataset size between ImageNet and UCF-101 was considerable. Furthermore, even though our result with HMDB-51 was lower than [13], please note that our result was higher by 0.85% compared with our baseline implementation.

### 5.4. System Limitations

Although our work improved the performance of action recognition compared to previous studies, there are limitations as follows. Firstly, when I3D was used as a backbone network, the absolute improvement was less than 1%. However, the state-of-the-art I3D baseline [13] was already at about 97.6% and 80%, so there was not enough room to improve. Therefore, the improvement may seem relatively low. Please note that the performance improvement was much larger compared to C3D [12] (6.84%). Accordingly, improving performance beyond such well-established works can be hard and valuable. We believe that our study is valuable in that it improved the performance by using new context cues different from other studies. Secondly, as shown in Table 4, the performance improvement on UCF-101 was 0.42%, and the performance was not improved on HMDB-51. However, this is due to the limitation of our re-implementation. As shown in Table 2 and Table 4, the reproduction results were 0.27% lower (97.6% vs. 97.33%) for the UCF-101 dataset and 1.23% lower (81.3 vs. 80.07) for the HMDB-51 dataset. We consider that this is because we cannot completely reuse the hyperparameters or GPU resources of the original baseline methods. Compared with the re-implementation results of this paper, the proposed method showed 0.69% and 0.85% improvement in UCF-101 and HMDB-51, respectively.

## 6. Conclusions

This paper proposed a multi-stream network based on contextual cues, which is generally available in the visual sensor-based action recognition algorithm. The proposed method does not require additional object location or human interaction annotation, which are not included in action recognition datasets. Furthermore, the proposed mask-based pairwise stream showed improved performance compared to the conventional bounding box-based method. In addition to the pairwise stream, we proposed to use the pose stream, which uses keypoints images. The proposed method outperformed both the well-known C3D network and the state-of-the-art I3D network. Furthermore, it improved the performance on two action recognition datasets, UCF-101 and HMDB-51.

## Figures and Tables

**Table 1:** Pairwise stream performance comparison.

Accuracy (%)	Bounding Box	Mask
Pairwise stream	49.80	**76.02**
Fusion	97.83	**98.02**

**Table 2:** The result of using C3D [12] and inflated 3D ConvNet (I3D) [13] as backbone networks for the UCF-101 and HMDB-51 datasets.

Method	UCF-101	HMDB-51
C3D-RGB—(our implementation)	84.17	-
C3D-Pose	80.35	-
C3D-Pairwise	79.87	-
C3D-(RGB and Pairwise and Pose)	**91.04**	-
I3D-RGB	94.69	74.84
I3D-Flow	94.14	77.52
I3D-Pose	69.15	51.57
I3D-Pairwise	76.02	51.83
I3D-(RGB and Flow)—(our implementation)	97.33	80.07
I3D-(RGB and Flow and Pairwise)	97.46	80.33
I3D-(RGB and Flow and Pose)	97.89	80.85
I3D-(RGB and Flow and Pairwise and Pose)	**98.02**	**80.92**

**Table 3:** The result of the UCF-101 dataset class accuracy of the baseline (I3D using RGB and optical flow) and proposed method.

Class	Baseline	Proposed (Improved)
HandstandPushups	82.14	98.43 (+14.29)
HandstandWalking	82.35	91.18 (+8.82)
CricketShot	89.80	95.92 (+6.12)
FrontCrawl	91.89	97.30 (+5.41)
Punch	89.74	94.87 (+5.13)
Shotput	93.48	97.83 (+4.35)
BoxingPunchingBag	73.47	77.55 (+4.08)
PullUps	96.43	100.00 (+3.57)
BodyWeightSquats	96.67	100.00 (+3.33)
HammerThrow	82.83	85.86 (+3.03)
FloorGymnastics	91.67	94.44 (+2.78)
WalkingWithDog	94.44	97.22 (+2.78)
Archery	95.12	97.56 (+2.44)
SoccerPenalty	97.56	97.22 (+2.78)
BaseballPitch	90.70	93.02 (+2.33)
PlayingCello	97.73	100.00 (+2.27)

**Table 4:** Comparison with other models.

Model	UCF-101	HMDB-51
LSTM (as reported in [13])	86.8	49.7
3D-ConvNet (as reported in [13])	79.9	49.4
Convolutional Two-Stream Network [33]	90.4	58.63
3D-Fused (as reported in [13])	91.5	66.5
Temporal Segment Networks [18]	93.5	-
Spatiotemporal Multiplier Networks [34]	94.0	69.02
Two-Stream I3D [13]	97.6	**81.3**
Multi-stream I3D (Proposed)	**98.02**	80.92
LSTM	91.0	53.4
Two-Stream	94.2	66.6
3D-Fused	94.2	71.0
Two-Stream I3D	98.0	81.2
